# L-shaped association of serum 25-hydroxyvitamin D with all-cause and cardiovascular mortality in older people with chronic kidney disease: results from the NHANES database prospective cohort study

**DOI:** 10.1186/s12889-023-16165-x

**Published:** 2023-06-28

**Authors:** Rugang Li, Yang Li, Zhongcheng Fan, Zhaoqi Liu, Juhua Lin, Min He

**Affiliations:** 1 Department of Nephrology, Affiliated Yuebei People’s Hospital of Shantou University Medical College, No. 133 South Huimin Road, Shaoguan, 512026 Guangdong China; 2 Department of Nephrology, Haikou Municipal People’s Hospital and Central South University Xiangya Medical College Affiliated Hospital, Haikou, China; 3 Department of Osteology, Haikou Municipal People’s Hospital and Central South University Xiangya Medical College Affiliated Hospital, Haikou, China; 4 Guangdong Medical University, Zhanjiang, 524001 Guangdong China

**Keywords:** Chronic kidney disease, 25-hydroxyvitamin D, Mortality, Cardiovascular diseases, Older people

## Abstract

**Background:**

This study was conducted to assess the association of serum 25-hydroxyvitamin D [25(OH)D] concentrations with all-cause and cardiovascular disease (CVD) mortality in older people with chronic kidney disease (CKD) in the United States.

**Methods:**

We identified 3230 CKD participants aged ≥ 60 years from the National Health and Nutrition Examination Survey (2001–2018). CKD was defined as an estimated glomerular filtration rate (eGFR) < 60 ml/min/1.73 m^2^. Mortality outcomes were determined by linkage to National Death Index (NDI) records through December 31, 2019. Restricted cubic spline based on Cox regression models were utilized to elucidate the nonlinear relationship between serum 25(OH)D concentrations and mortality in patients with CKD.

**Results:**

During median 74 months of follow-up, 1615 all-cause death and 580 CVD death were recorded. We found an L-shaped association between serum 25(OH)D concentrations and all-cause and CVD mortality, reaching a plateau at 90 nmol/L. Accordingly, per one-unit increment in natural log-transformed 25(OH)D was associated with a 32% and 33% reduced risk of all-cause mortality (hazard ratio [HR] 0.68; 95%CI, 0.56 to 0.83) and CV mortality (HR 0.69; 95%CI, 0.49 to 0.97) in participants with serum 25(OH)D < 90 nmol/L, but no considerable difference was observed in participants with serum 25(OH)D ≥ 90 nmol/L. Compared with those in the deficiency group (< 50 nmol/L), insufficient (50 to < 75 nmol/L) and sufficient group (≥ 75 nmol/L) were significantly associated with lower all-cause mortality (HR,0.83; 95%CI, 0.71 to 0.97 and HR, 0.75; 95%CI, 0.64 to 0.89) and CV mortality (HR,0.87; 95%CI, 0.68 to 1.10 and HR, 0.77; 95%CI, 0.59 to < 1.0), respectively.

**Conclusion:**

An L-shaped relationship between serum 25(OH)D levels with all-cause and CVD mortality was observed in elderly CKD patients in the United States. A 25(OH)D concentration of 90 nmol/L may be the target to reduce the risk of premature death.

## Background

Chronic kidney disease (CKD) affects more than 10% of the general population worldwide, and this situation is more common among older people [1, 2]. CKD has become one of the major causes of death from non-communicable diseases worldwide [3]. It is critical to identify modifiable factors to prevent premature death from CKD in older adults.

25-hydroxyvitamin D [25(OH)D] is the main storage form of vitamin D and is involved in phosphate and calcium metabolism. Vitamin D deficiency is associated with musculoskeletal health, cardiovascular diseases, hypertension, autoimmune diseases, obesity, diabetes and depression [4–7]. A single-center retrospective study and post-hoc subgroup analysis of the LURIC study both found that low 25(OH)D levels were associated with increased all-cause mortality [8, 9]. Additionally, a meta-analysis of 10 studies found that higher 25(OH)D levels were associated with better survival in patients with CKD [10]. The study of NHANES III (Third National Health and Nutrition Examination Survey 1988–1994) showed non-dialysis CKD patients with low serum 25(OH)D levels had an increased risk of death compared to the high level group [11]. Lifestyles, the spectrum of disease, and treatments have dramatic changes during the last two decades. It is necessary to re-assess the relationship between 25(OH)D and risk of death. Additionally, the effectiveness of vitamin D supplementation on CVD and all-cause mortality is controversial. This could be attributed to uncertainty about the optimal serum 25(OH)D concentration necessary for intervention. Given that most published studies are small, relatively old data, and inadequate adjustments for important factors in CKD, further studies are needed to investigate the optimal vitamin D concentration for patients with CKD is crucial.

We conducted a large prospective cohort study, based on the National Health and Nutrition Examination Survey (NHANES) to assess the association between serum 25(OH)D levels with all-cause and CVD mortality, and to evaluate the optimal target level of vitamin D supplementation in elderly patients with CKD.

## Methods

### Study design and population

NHANES is a complex, multistage sampling design nationally representative study to assess the health and nutritional status of the noninstitutionalized civilian population in the U.S. From 1999, it became a continuous program, with every two years representing a cycle [12]. NHANES was performed by the Centers for Disease Control and Prevention (CDC), and approved by the institutional review board of the National Center of Health Statistics. All participants provided written informed consent.

We used nine cycles of the NHANES from 2001 to 2018 (vitamin D data were not available in the NHANES 1999–2000). There are 17,222 individuals aged ≥ 60 years who are eligible for follow up. 3646 participants with CKD diagnosis [estimated glomerular filtration rate (eGFR) < 60 ml/min/1.73 m^2^] and not dialysis in the previous 12 months were included in this analysis. Those with missing information on age, sex, race, education, BMI, biochemical test, comorbidities, co-medications (*n* = 354), or abnormal values (*n* = 62) were further excluded. Therefore, a total of 3230 participants entered in our present analysis.

### Measurement of Serum 25(OH)D

In NHANES, the concentration of serum 25(OH)D was determined by DiaSorin radioimmunoassay kit (Stillwater, MN) in 2001–2006, and subsequently by standardized liquid chromatography-tandem mass spectrometry (LC–MS/MS) since the 2007–2008 cycle. In accordance with CDC recommendations, we used LC–MS/MS equivalent data for all analyses. The details of the conversion are specified elsewhere [13].

### Ascertainment of mortality

Mortality from any cause and CVD was ascertained by linkage to the National Death Index through 31 December 2019. The ICD-10 was used to determine disease-specific death. CVD mortality was defined as the primary cause of death being any disease of the circulatory system (ICD-10 codes I00-I09, I11, I13, I20-I51, or I60–I69).

### Assessment of covariates

Information on age, sex, race, education level, smoking status, recreational activity, comorbidities, and medication use was collected from household interviews using standardized questionnaires. Body weight, height were obtained when people participated in physical examinations at a mobile examination center. BMI was calculated as weight in kilograms divided by height in meters squared. Race was classified as non-Hispanic White, non-Hispanic Black, Mexican American, or other. Education level was categorized as less than high school, high school or equivalent, or college or above. Smoking status was classified as never smoker, former smoker, or current smoker. Physical activity was defined based on the three common domains of participation corresponding to the interviewer-administered Physical Activity Questionnaire (PAQ), including leisure and recreational activities, occupation-related physical activity, and transportation-related physical activity. This study focused only on leisure and recreational activities and defined as “yes” as long as participation in the activities [14].

In addition, creatinine, triglycerides, total cholesterol, UACR, and parathyroid hormone (PTH) were measured at baseline when the participants provided their blood samples. eGFR was calculated by the CKD Epidemiology Collaboration equation [15].

Diabetes was defined as self-reported doctor diagnosis of diabetes, use of insulin or oral hypoglycemic medication, fasting glucose ≥ 7.0 mmol/L, random glucose ≥ 11.1 mmol/L, or glycated hemoglobin A1c (HbA1c) ≥ 6.5%. Hypertension status was obtained from self-report, or systolic blood pressure ≥ 140 mmHg or diastolic blood pressure ≥ 90 mm Hg. CVD contains self report coronary heart disease, congestive heart failure, heart attack, stroke, and angina. CCI was calculated based on the literature published in 1994 [16].

### Statistical analysis

Sample weights, clustering, and stratification were incorporated into all analyses because of the complex sampling design of the NHANES. Participants were followed up to death or the date of 31 December 2019, whichever comes first. According to the Endocrine Society Clinical Practice Guidelines, vitamin D status was categorized into three groups: deficiency (< 50 nmol/L), insufficient (50 to < 75 nmol/L), and sufficient (≥ 75 nmol/L) [17]. Serum 25(OH)D concentrations were also analyzed as a continuous variable after natural log transformation. We estimated the relationship between serum 25(OH)D concentrations and all-cause mortality as well as CVD mortality using Cox proportion hazard model, with or without adjustment for age, sex, age, race, education, smoking status, BMI, and recreational activity, UACR, eGFR, total cholesterol, triglycerides, comorbidities (hypertension, diabetes, CVD, CCI), and co-medications. Additionally, a restricted cubic spline based on Cox regression, with 3 knots, was performed to test for linearity and explore the shape of the dose–response relation of serum 25(OH)D concentrations and mortality.

Stratified analyses were also conducted by sex, ethnicity (White or non-White), BMI (< 30.0, or ≥ 30.0 kg/m^2^), eGFR(< 45, ≥ 45 ml/min/1.73m^2^), and with or without CVD, hypertension and diabetes.

Two sensitivity analyses were performed to test the robustness of our findings. First, to reduce the potential reverse causal bias, we included participants with more than 2 years of follow-up. Second, considering the interrelationship of PTH and vitamin D status, PTH was further adjusted (only available in the NHANES 2003–2006, *n* = 737).

All analyses were carried out with R version 4.2.0, and a two-tailed *P* < 0.05 was considered statistically significant.

## Results

Among the 3230 elderly CKD participants (mean age, 74.5 years; 52.1% female), 732 (23.9%) had deficient vitamin D (< 50 nmol/L) and 1061 (32.8%) had insufficient vitamin D (< 75 nmol/L). The baseline characteristics of the study population according to serum 25(OH)D status are shown in Table 1. Participants who had higher 25(OH)D levels were more likely to be female, non-Hispanic White; had higher education levels and recreational activity; were less likely to be current smoker and obese; and lower comorbidities.

**Table 1:** Baseline characteristics of participants with CKD stratified by serum 25(OH)D concentrations

Variables^a^	Serum 25(OH)D concentrations (nmol/L)^b^			*P* value
	** < 50 (*n* = 772)**	**50 to < 75 (*n* = 1061)**	** ≥ 75 (*n* = 1397)**	
Age, yr	73.9 (0.3)	74.5 (0.3)	74.6 (0.2)	0.21
Female, n (%)	451 (67.6)	494 (53.6)	739 (59.8)	< 0.001
Race, n (%)				< 0.001
White	357 (70.1)	721 (84.3)	1019 (87.6)	
Black	233 (17.5)	146 ( 6.8)	185 ( 5.6)	
Mexican American	99 (4.1)	84 (2.7)	65 (1.7)	
Other	83 (8.3)	110 (6.2)	128 (5.2)	
Education, n (%)				< 0.001
Low than high school	332 (34.5)	371 (27.2)	360 (19.4)	
High school	182 (27.0)	274 (28.2)	374 (27.7)	
College	258 (38.6)	416 (44.7)	663 (52.9)	
Smoke, n (%)				< 0.001
Never	373 (48.1)	506 (49.5)	692 (52.2)	
Former	297 (37.7)	466 (43.0)	617 (42.7)	
Current	102 (14.2)	89 ( 7.5)	88 ( 5.2)	
BMI, kg/m^2^				< 0.001
< 25	176 (22.3)	250 (22.6)	376 (26.2)	
25–29	240 (29.8)	400 (37.0)	558 (38.5)	
≥ 30	356 (48.0)	411 (40.4)	463 (35.3)	
Recreational activity, n (%)	109 (13.4)	248 (25.6)	455 (36.3)	< 0.001
eGFR, ml/min/1.73m^2^	46.7 (0.5)	48.49 (0.4)	48.0 (0.3)	0.01
UACR, mg/g	263.5 (46.1)	115.9 (18.0)	75.0 ( 9.6)	< 0.001
TCHO, mmol/L	4.9 (0.1)	5.0 (0.0)	4.9 (0.1)	0.12
TG, mmol/L	1.8 (0.1)	1.9 (0.1)	1.8 (0.0)	0.31
CCI, count	2.5 (0.1)	2.1 (0.1)	2.4 (0.1)	0.003
Hypertension, n (%)	655 (83.3)	843 (78.2)	1137 (80.3)	0.1
Diabetes, n (%)	348 (44.2)	388 (32.8)	491 (31.3)	< 0.001
CVD, n (%)	321 (42.9)	421 (37.5)	516 (34.7)	0.01
Anti-hypertension, n (%)	579 (76.3)	751 (68.2)	1014 (68.8)	0.01
Lipid-lowering drugs, n (%)	347 (48.2)	503 (45.9)	764 (55.0)	< 0.001
Hypoglycemic, n (%)	239 (29.8)	271 (23.6)	335 (21.6)	0.002

*Abbreviation*: *BMI* Body mass index, *UACR* Urinary albumin creatinine ratio, *eGFR* Estimated glomerular filtration rate, *TCHO* Total cholesterol, *TG* Triglycerides, *CVD* Cardiovascular disease, *CCI* Charlson Comorbidity Index

^a^All estimates accounted for complex survey designs. Continuous variables were expressed as mean (standard error). Categorical variables were expressed as number (percent)

^b^All intervals are left closed and right open

**Fig. 1:**
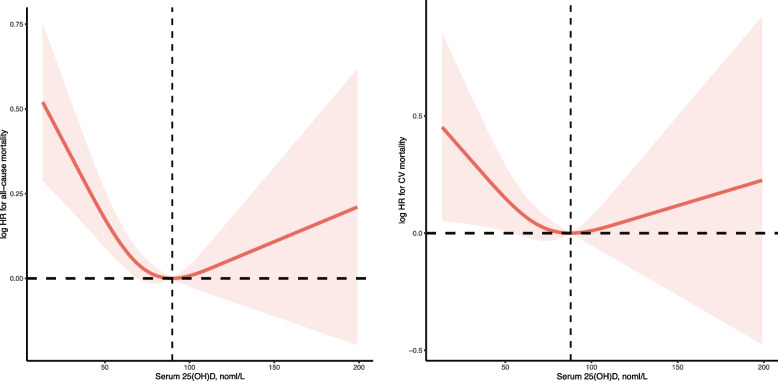
Relation of serum 25(OH)D concentrations with all-cause and CVD mortality. Hazard ratio (HR) was adjusted for age, sex, race, education, smoking status, BMI, recreational activity, UACR, eGFR, total cholesterol, triglycerides, comorbidities (hypertension, diabetes, CVD, CCI), and co-medications. Abbreviation: CVD, Cardiovascular disease

During a median follow-up of 74 months (interquartile range, 38–119 months), 1615 all-cause deaths were documented, including 580 CV deaths. The association between serum 25(OH)D concentrations (Fig. 1) with the risk of all-cause and CV mortality followed a L-shape (P for nonlinearity 0.002 and 0.01), with the smallest Akaike information criterion (AIC) of three knots. Accordingly, in the threshold effect analysis, per one-unit increment in natural log-transformed 25(OH)D was associated with a 32% and 33% reduced risk of all-cause mortality (hazard ratio [HR] 0.68; 95%CI, 0.56 to 0.83) and CV mortality (HR 0.69; 95%CI, 0.49 to 0.97) in participants with serum 25(OH)D < 90 nmol/L, but no considerable difference was observed in participants with serum 25(OH)D ≥ 90 nmol/L (Table 2).

**Table 2:** Threshold effect analyses of serum 25(OH)D with all-cause and CVD mortality using two-piecewise regression models

Serum 25(OH)D, nmol/L	Crude HR (95%CI)^a^	Adjusted HR (95%CI)^ab^
< 90 (*n* = 2381)		
All-cause mortality	0.62 (0.54,0.72)	0.68 (0.56,0.83)
CVD mortality	0.67 (0.50,0.88)	0.69 (0.49,0.97)
≥ 90 (*n* = 849)		
All-cause mortality	0.83 (0.31,2.23)	1.43 (0.55,3.72)
CVD mortality	1.18 (0.30,4.62)	2.66 (0.68,10.44)

*Abbreviation*: *CVD* Cardiovascular disease

^a^Per one-unit increment in natural log-transformed 25(OH)D

^b^Adjusted for age, sex, race, education, smoking status, BMI, recreational activity, UACR, eGFR, total cholesterol, triglycerides, comorbidities (hypertension, diabetes, CVD, CCI), and co-medications

In addition, we also included the serum 25(OH)D concentrations as a categorical variable divided into three levels, deficiency (< 50 nmol/L), insufficient (50 to < 75 nmol/L) and sufficient (≥ 75 nmol/L), and the sufficient category including the inflection point (90 nmol/L). Compared with those in the deficiency group, the insufficient and sufficient groups were significantly associated with lower all-cause mortality (HR,0.83; 95%CI, 0.71 to 0.97 and HR, 0.75; 95%CI, 0.64 to 0.89) and CV mortality (HR,0.87; 95%CI, 0.68 to 1.10 and HR, 0.77; 95%CI, 0.59 to < 1.0), respectively (Table 3).

**Table 3:** The association between serum 25(OH)D concentrations with all-cause and CVD mortality

	**Serum 25(OH)D concentrations (nmol/L)**		
	** < 50 (*n* = 772)**	**50 to < 75 (*n* = 1061)**	** ≥ 75 (*n* = 1397)**
All-cause mortality			
Number of deaths	485	593	537
Crude HR (95%CI)	reference	0.79 (0.69,0.90)	0.68 (0.59,0.79)
Adjusted HR (95%CI)^a^	reference	0.83 (0.71,0.97)	0.75 (0.64,0.89)
CVD mortality			
Number of deaths	164	228	188
Crude HR (95%CI)	reference	0.86 (0.68,1.09)	0.69 (0.54,0.88)
Adjusted HR (95%CI)^a^	reference	0.87 (0.68,1.10)	0.77 (0.59, < 1.0)

*Abbreviation*: *CVD* Cardiovascular disease

^a^Adjusted for age, sex, race, education, smoking status, BMI, recreational activity, UACR, eGFR, total cholesterol, triglycerides, comorbidities (hypertension, diabetes, CVD, CCI), and co-medications

**Fig. 2:**
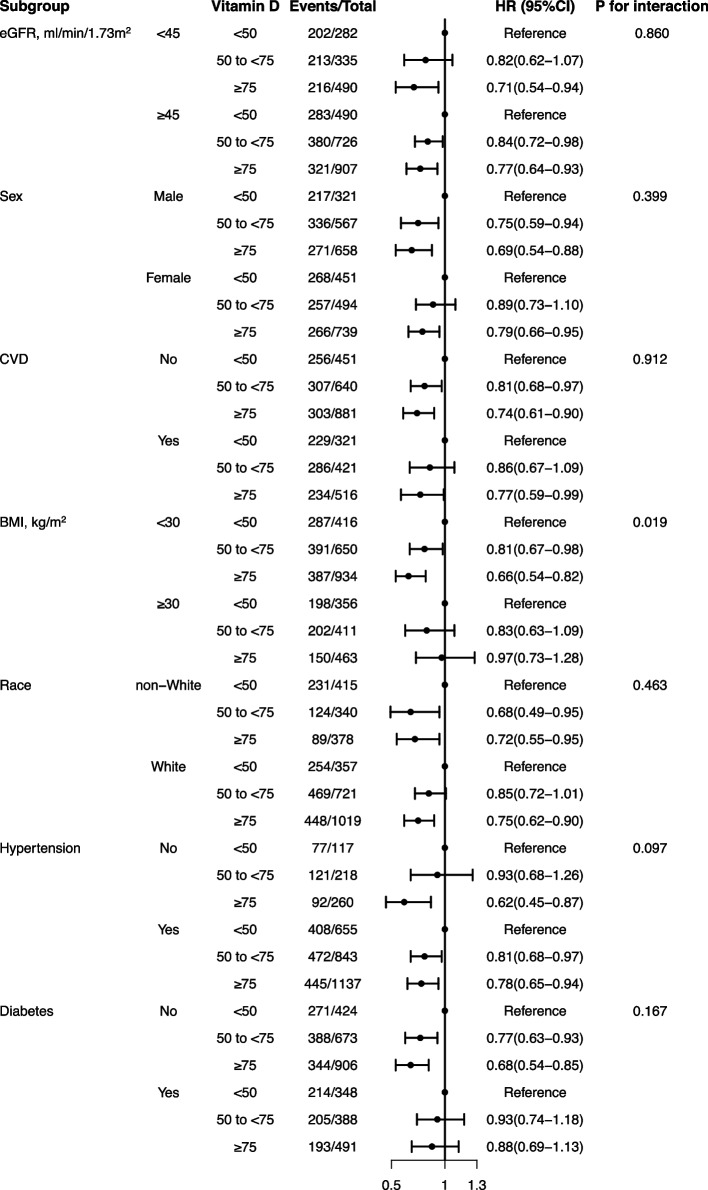
Association between serum 25(OH)D concentrations and all-cause mortality in various subgroups. Hazard ratio (HR) was adjusted for age, sex, race, education, smoking status, BMI, recreational activity, UACR, eGFR, total cholesterol, triglycerides, comorbidities (hypertension, diabetes, CVD, CCI), and co-medications. Abbreviation: eGFR, estimated glomerular filtration rate; CVD, Cardiovascular disease

In the subgroup analysis, the association between 25(OH)D and all-cause mortality was consistent across subgroups stratified by sex, race, eGFR, presence of CVD, hypertension and diabetes. Although, the interactions for BMI were lower than 0.05, due to decreased tendency in the similar directionality of the associations in those with BMI more than 30 kg/m^2^, suggesting that there are more factors related to death in obese people besides vitamin D (Fig. 2).

In the sensitivity analyses, compared with the deficiency level, the results of the insufficient group (HR,0.76; 95%CI, 0.59 to 0.99) and sufficient group (HR,0.65; 95%CI, 0.47 to 0.92) did not significantly change when we included participants with more than 2 years of follow-up. Similarly, when further adjusted for PTH, the results also did not materially change (HR, 0.74; 95%CI, 0.57 to 0.95) and (HR,0.68; 95%CI, 0.49 to 0.94).

## Discussion

This large prospective cohort study of US older people with CKD showed a L-shaped association between serum 25(OH)D concentrations with all-cause and CVD mortality, with the plateau at 90 nmol/L. This association was independent of traditional risk factors, and was consistently observed across subgroups stratified by eGFR (< 45, ≥ 45 ml/min/1.73m^2^), gender, BMI (< 30, ≥ 30 kg/m^2^), race (White, non-White) and with or without CVD, diabetes, and hypertension.

The relationship between 25(OH)D concentration and mortality remains a topic of interest to clinicians and the public in patients with CKD. Our study showed a high prevalence of insufficient 25(OH)D (32.8%) in patients with CKD, indicating widespread vitamin D deficiency in those people. Consistent with previous research [8, 10, 11, 18], our multivariate analysis showed that higher serum 25(OH)D concentrations were independently associated with reduced all-cause and cardiovascular mortality.

The definition of sufficiency and toxicity thresholds for vitamin D remain unclear [19]. A retrospective cohort study showed an independent association between 25(OH)D levels of less than 37.5 nmol/L and all-cause mortality in non-dialysis CKD patients [8]. In a previous NHANES study [11], subgroups with 25(OH)D levels less than 37.5 nmol/L had a higher risk of all-cause mortality compared to subgroups with 25(OH)D levels higher than 75 nmol/L with or without multiple adjustment. We prepared serum 25(OH)D < 50 nmol/L as the reference group, and a significant reduction of all-cause and CVD mortality in the 50–75 nmol/L group and ≥ 75 nmol/L group was observed. Moreover, we observed an L-shaped relationship between serum 25(OH)D concentration with all-cause and CVD mortality, similar to a large general population survey in the United Kingdom [20]. Their study showed an L-shaped relationship between genetically predicted 25-(OH)D and all-cause mortality, with a sharp increase risk of death at concentrations below 50 nmol/L. In another cohort study (UK Biobank, EPIC-CVD and two Copenhagen population studies) utilizing population-based Mendelian randomization analysis also showed a non-linear dose–response relationship of vitamin D with coronary heart disease, stroke and all-cause mortality [21]. They observed an inverse correlation between genetically predicted 25(OH)D concentrations and all-cause mortality up to 40 nmol/L. This association is usually nonlinear. Mortality decreases with increasing concentrations of 25(OH)D until a certain inflection point, beyond which further mortality declines are not observed. An inflection point at 90 nmol/L was observed in this older CKD population, beyond which no further reduction of death was shown. The finding suggest that vitamin D requirements is differs in various disease, and older patients with CKD may need relatively higher levels of vitamin D. Mendelian randomization studies require consideration of instrumental variables. Because genes are randomized and polymorphic, this may lead to large differences in study results across populations. However, our research is a nationally representative survey with a complex, multi-stage sampling design. The variables are simple, standardized and comprehensive, therefore the findings of the study may be more robust and representative.

There is few evidence shown beneficial effects of vitamin D supplement on clinical outcomes. In the general population, vitamin D supplementation has not significantly reduced mortality from all-causes or CVD [22, 23]. One possibility is that people with vitamin D deficiency or severe deficiency were excluded from the study. Furthermore, the effects of vitamin D supplement on all-cause and CVD mortality in CKD individuals are controversial [19, 24]. This may be because there is no further reduction in mortality when serum vitamin D concentrations reach a certain threshold. Our analysis of data from a nationally representative population-based survey showed an L-shaped relationship between serum 25(OH)D concentrations and all-cause and CVD mortality, which could partially explain these controversies. Future randomized controlled trials need to fully account for this L-shaped relationship.

There are several possible mechanisms explaining the association between lower 25(OH)D levels and an increased risk of mortality. Numerous diseases that cause death have been linked to low vitamin D levels, such as dyslipidemia, hypertension, and diabetes [25–27]. Moreover, low vitamin D levels are associated with coronary artery calcification, thickening of the carotid intima-media and other cardiovascular disease [28–30]. These conditions may therefore constitute a risk of mortality, while high levels of vitamin D may reduce signal between renin, angiotensin and aldosterone, improve endothelial dysfunction and modulate immune function [31–33]. By inhibiting macrophage migration and cholesterol uptake, suppressing foam cell formation, and reversing atherogenic cholesterol metabolism, it attenuates atherosclerosis [34, 35]. There is evidence that secondary hyperparathyroidism associated with low vitamin D levels increases all-cause and CVD mortality in multiple ways. In addition, it activates the RAS system, worsens hypertension, and exacerbates anemia by exacerbating abnormalities in calcium and phosphorus metabolism and thus vascular calcification [36–38]. Nevertheless, further mechanistic studies are needed to better understand how vitamin D prevents all-cause and CVD mortality in patients with CKD.

Strengths of our study include the prospective study design, relatively large sample size, and a nationally representative sample of older Americans with CKD, which facilitates replication. As well, this study had sufficient statistical power due to the substantial number of deaths during a long period of time. Moreover, we adjusted for multiple potential confounders based on the understanding of elderly population, that improved the validity and robustness of our conclusions. There are also some limitations to be considered. First, because it was an observational study, residual confounding factors are possible. Second, serum 25(OH)D concentrations were measured only once, which may underestimate the true association of interest [39]. Third, the current study did not assess vitamin D metabolite ratios (e.g., the ratio of serum 25[OH]D to 1,25[OH]2D could be used as a surrogate for 24-hydroxylase) and genetic variation in genes related to vitamin metabolism [19]. Third, this study defined CKD as eGFR < 60 ml/min/1.73 m^2^. It should be cautious when generalizing the finding to patients with albuminuria and hematuria alone. Finally, the role of psychosocial stress or genetic susceptibility cannot be excluded in the current study.

## Conclusions

In a nationally representative sample of older people with CKD, we found a L-shaped association between serum 25(OH)D concentrations with all-cause and CVD mortality, with the plateau at 90 nmol/L. The threshold of 90 nmol/L may be a target for interventions to reduce the risk of death.

## Data Availability

The datasets generated and analyzed in the current study are available at NHANES website: https://www.cdc.gov/nchs/nhanes/index.htm.

## Abbreviations

CKD: Chronic kidney disease
CVD: Cardiovascular disease
25(OH)D: 25-Hydroxyvitamin D
NHANES: National Health and Nutrition Examination Survey
NDI: National Death Index
NHANES III: Third National Health and Nutrition Examination Survey
CDC: Centers for Disease Control and Prevention
95% CI: 95% Confidence interval
eGFR: Estimated glomerular filtration rate
HR: Hazard ratio
PIR: Family poverty income ratio
BMI: Body mass index
UACR: Urinary albumin creatinine ratio
eGFR: Estimated glomerular filtration rate
TCHO: Total cholesterol
TG: Triglycerides
CVD: Cardiovascular disease
CCI: Charlson Comorbidity Index
